# Effect of Pregnane X Receptor^*^1B genetic polymorphisms on postoperative analgesia with fentanyl in Chinese patients undergoing gynecological surgery

**DOI:** 10.1186/s12881-016-0348-5

**Published:** 2016-11-23

**Authors:** Jing-Jing Yuan, Xiao-Jing Ma, Zhi-Song Li, Yan-Zi Chang, Wei Zhang, Quan-Cheng Kan, Jun-Kai Hou, Li-Rong Zhang

**Affiliations:** 1 Department of Anesthesiology, First Affiliated Hospital of Zhengzhou University, No.1 East-JianShe Road, Zhengzhou, 450052 China; 2 Key Laboratory of Pharmacogenomics, No.1 East-JianShe Road, Zhengzhou, Henan Province 450052 China; 3 First Affiliated Hospital of Zhengzhou University, No.1 East-JianShe Road, Zhengzhou, 450052 China; 4 Department of Pharmacology, School of Medicine, Zhengzhou University, Zhengzhou, 450000 China

**Keywords:** Fentanyl, CYP3A4, Pregnane X receptor, Polymorphism, Analgesia

## Abstract

**Background:**

The purpose of the study was to investigate the effects of the pregnane X receptor (PXR)*1B polymorphisms on CYP3A4 enzyme activity and postoperative fentanyl consumption in Chinese patients undergoing gynecological surgery.

**Methods:**

A total of 287 females of Han ethnicity, aged 20 to 50 years old, ASA I or II, scheduled to abdominal total hysterectomy or myomectomy under general anesthesia were enrolled. The analgesic model used was fentanyl consumption via patient-controlled intravenous analgesia (PCIA) in the post-operative period. Additionally, pain was assessed using a visual analog score (VAS). Pain scores, occurrence of adverse reactions and consumption of fentanyl were recorded during the 24 h postoperative period. The enzyme activity of CYP3A4 was evaluated by measuring the plasma ratio of 1′-hydroxymidazolam to midazolam 1 h after intravenous administration of 0.1 mg/kg midazolam. PXR genotyping was performed by direct DNA sequencing and the *PXR* ^*^ *1B* haplotype was analyzed via PHASE V.2.1 software.

**Results:**

The polymorphism frequency of *PXR11156A* > *C/11193 T > C and* 8055*C > T* were 49.6 and 49.3%, and the rate of *PXR* ^*^ *1B* haplotype was 48.8% in our study. None of the pain scores, consumption of fentanyl 24 h post-operatively or enzyme activity of CYP3A4, showed differences among different genotypes.

**Conclusions:**

*PXR11156A > C*, *PXR11193T > C*, *PXR8055C > T* or the *PXR* ^*^ *1B* haplotype do not appear to be important factors contributing to CYP3A4 activity and interindividual variations in postoperative fentanyl consumption in Han female patients undergoing gynecological surgery.

**Trial registration:**

The DNA samples were obtained since 2007 to 2010 year in our hospital, there was no registration at that time. So this section is not applicable to our research.

## Background

Fentanyl, an intravenous μ-opioid receptor agonist, is widely used for postoperative analgesia. However, the analgesic effect of fentanyl demonstrates significant inter-individual variation [1]. These variations in response can lead to inadequate pain relief and may affect the incidence of adverse events such as respiratory depression and muscle stiffness [2, 3]. Dolin et al. [4] reported that approximately 30–40% of patients using postoperative patient-controlled analgesia (PCA) reported moderate to severe pain and 10% reported severe pain.

Part of the variable response to pain medications may be attributed to genetic factors [5].

Fentanyl is mainly metabolized by cytochrome P450 family 3 subfamily A member 4, (CYP3A4) [6–8], which is the most abundant metabolic enzyme in liver. The level of expression and activity of CYP3A4 showed a 10–100-fold inter-individual difference [9–11]. Prior studies have indicated that genetic factors could significantly affect the pharmacokinetics and pharmacodynamics of drugs by altering the expression of drug-metabolizing enzymes or their activities [12, 13].

PXR is a nuclear receptor that serves a primary role in the up-regulation of proteins and enzymes in response to endogenous and exogenous substances and it was found to be a key regulator of CYP3A4 [11, 12]. Polymorphic variations of the PXR gene, such as the haplotype of *PXR* ^*^ *1B* has been confirmed to contribute to both of the mRNA level and the protein content of CYP3A4 [13, 14]. The *PXR* ^*^ *1B* haplotype is composed of *PXR 11193 T > C* and *PXR 8055C > T*, *PXR 11156A > C* is in complete linkage with *PXR 11193 T > C* and is similar to *it* [14, 15]. PXR*1B has three haplotypes: *non-PXR* ^*^ *1B* haplotype (wild homozygotes of the three genes), *PXR* ^*^ *1B* haplotype (heterozygotes of the three genes) and *PXR* ^*^ *1B/* ^*^ *1B* (mutant homozygotes of the three genes). However, it is still elusive if *PXR* ^*^ *1B* haplotype has sufficient effect on the catalytic activity of the CYP3A4 enzyme, and subsequently alter the analgesic effect of fentanyl for postoperative pain control.

## Methods

Some of methods were referred to the study done by W Zhang et al. [6] as the following:

### Subjects

A total of 287 females, of self-reported Han ethnicity, aged20 to 50 years old, with a physical status of American Society of Anesthesiologists (ASA) I or II and scheduled for abdominal total hysterectomy or myomectomy under general anesthesia were screened at the First Affiliated Hospital of Zhengzhou University. All subjects and genetic material obtained during the period of 2007 and 2010 were a subset of a larger group of 330 patients collected for medical studies. All samples that had relative full information and a usable blood sample were included in this study. The study protocol was previously approved by the Institutional Ethics Committee of Zhengzhou University and every patient provided signed informed consent. Participants with a history of psychiatric diseases, cardiovascular diseases, hepatic and renal dysfunction, alcohol or drug abuse, diabetes or chronic analgesic use were excluded. Patients were also questioned about diet and medication intake 2 weeks prior to their surgery and were excluded if they reported consuming any drugs or food that are known to inhibit the expression level or activity of the CYP3A4 enzyme (clarithromycin, erythromycin, grapefruit juice etc.) [6]. All patients were operated on by the same group of 4 surgeons.

### Anesthetic procedure

Patients were not given any premedication and standard monitors were used. During the induction of anesthesia, midazolam (0.1 mg/kg), propofol (0.5 mg/kg) and remifentanil (2 μg/kg) were utilized. Succinylcholine (1 mg/kg) provided muscle relaxation for intubation. During the surgical procedure, propofol 100–150 mcg/kg/min and remifentanil 0.1–0.2 μg/kg/min were continuously infused. To maintain adequate muscle relaxation, an initial dose of atracurium (0.6 mg/kg) was given immediately after tracheal intubation, and intermittent doses (0.1–0.2 mg/kg) were utilized during the case. All patients were intubated and mechanically ventilated with a target end-tidal CO_2_ of 35–40 mmHg. Fentanyl (1 μg/kg), as a loading dose, was infused intravenously approximately 30 min prior to the completion of surgery. All anesthetics were stopped at the end of surgery. Atropine (0.5 mg) and neostigmine (1 mg) were used to reverse muscle relaxation in all patients. Patients were extubated after adequate recovery as was judged by the anesthesiologist.

### Assessment of postoperative analgesia

After extubation, all patients were assessed in the operating room using a visual analog scale (VAS, 0 = no pain, 10 = unbearable pain). Fentanyl 20 μg was given to patients whose VAS was over 3 and was redosed every 5 minutes until the VAS score was less than 3; at which time a PCA with fentanyl was immediately begun. The dose of fentanyl, VAS score, the occurrence of adverse reactions after surgery and 24 h after surgery were recorded.

The PCA pump contained a total of fentanyl 1.0 mg, droperidol 5 mg and saline to reach a total volume of 100 ml, with a final concentration of 10 μg/ml of fentanyl. The pump was set with a background rate of 0.5 ml per hour (a background infusion of 5 μg fentanyl/h). Doses were set at 2 ml, with a lockout time of 5 min, allowing a maximum dose of 145 μg per hour. If the VAS score remained above 3, with a maximum dose of fentanyl, other analgesics would be given and the patient was be excluded from the trial.

### Genotyping for *PXR* polymorphisms

**Fig. 1:**
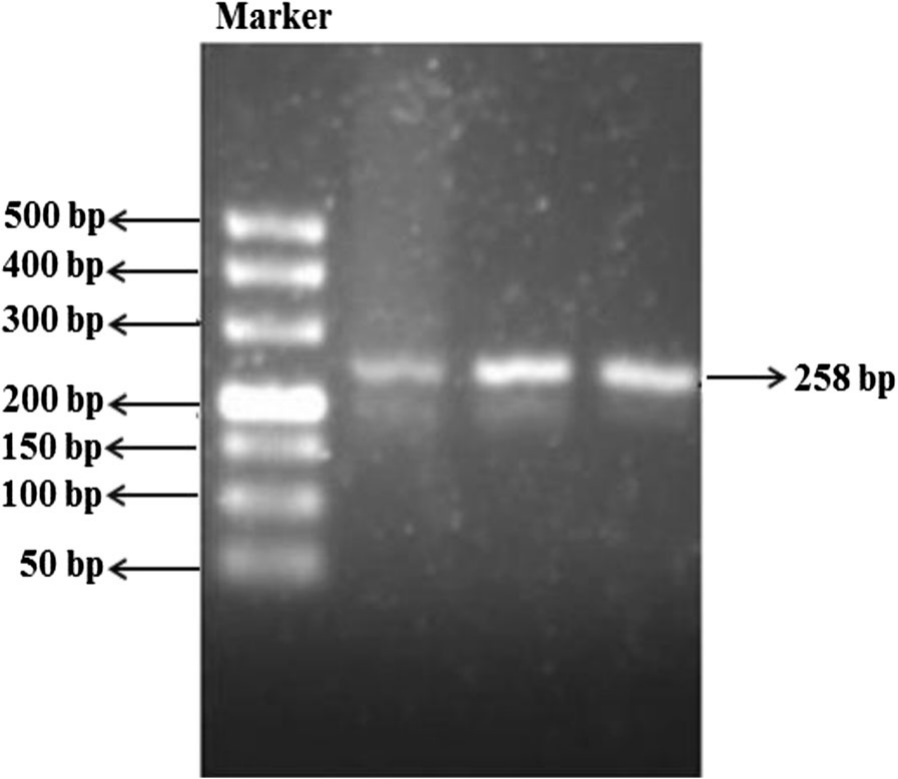
The electrophoregram of *PXR11156A > C* and *PXR11193T > C*. Peripheral venous blood samples were taken from all patients. Genomic DNA was extracted using a conventional phenol-chloroform procedure. Genotyping of *PXR11156A > C, PXR11193T > C* allele was conducted by polymerase chain reaction (PCR), following with direct sequencing. PXR*1B haplotype was genotyped by PHASE V.2.1 software. Figure 1 showed the 258 bp band of the PCR amplification product which contained *PXR11193T > C.* The DM500 marker, which contains the bands of 50 bp, 100 bp, 150 bp, 200 bp, 300 bp, 400 bp and 500 bp was used

**Fig. 2:**
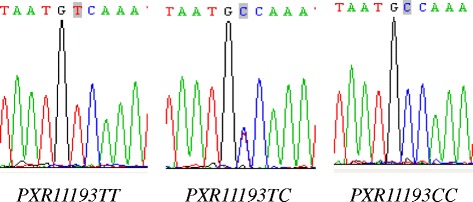
The DNA sequence of *PXR11193T > C.* Genomic DNA was extracted using a conventional phenol-chloroform procedure. Genotyping of *PXR11193T > C* allele was conducted by polymerase chain reaction (PCR), direct sequencing. PXR*1B haplotype was genotyped by PHASE V.2.1 software. Two T bases were showed in the *PXR11193T > C* wild homozygotes. One T base and one C base were showed in the *PXR11193T > C* heterozygotes. Two C bases were showed in the mutant homozygotes

**Fig. 3:**
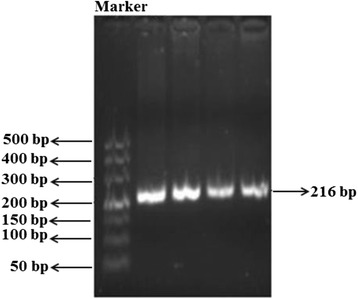
The electrophoresis of *PXR8055C > T.* Genomic DNA was extracted using a conventional phenol-chloroform procedure. Genotyping of *PXR8055C > T* allele was conducted by polymerase chain reaction (PCR), following with direct sequencing. PXR*1B haplotype was genotyped by PHASE V.2.1 software. Figure 3 showed the 216 bp band of the PCR amplification product which contained *PXR8055C > T.* The DM500 marker, which contains the bands of 50 bp, 100 bp, 150 bp, 200 bp, 300 bp, 400 bp and 500 bp was used

**Fig. 4:**
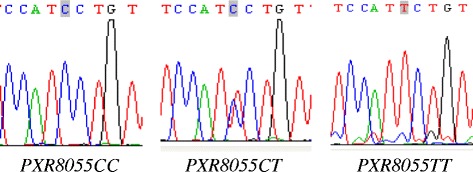
The DNA sequence of *PXR8055C > T.* Genomic DNA was extracted using a conventional phenol-chloroform procedure. Genotyping of *PXR8055C > T* allele was conducted by polymerase chain reaction (PCR), direct sequencing. PXR*1B haplotype was genotyped by PHASE V.2.1 software. Two C bases were showed in the *PXR8055C > T* wild homozygotes. One T base and one T base were showed in the *PXR8055C > T* heterozygotes. Two T bases were showed in the mutant homozygotes

Peripheral venous blood samples (2 ml) were obtained from all patients enrolled. Genomic DNA was extracted using a conventional phenol-chloroform procedure. Genotyping of *PXR11156A > C*, *PXR11193T > C* and *8055C > T* was conducted by polymerase chain reaction (PCR) and direct sequencing. The *PXR* ^*^ *1B* haplotype was genotyped by PHASE V.2.1 software. The results, including agarose gel electrophoresis and DNA sequencing, are shown in Figs. 1, 2, 3 and 4.

Genotyping of the *PXR11156A > C* and *PXR11193T > C* polymorphisms was previously described by Wang XD et al. [15]. Genotyping of the *PXR8055C > T* polymorphism was identified by the following process, a 216-bp fragment, including the single nucleotide polymorphism (SNP) *8055C > T,* was created. The forward and reverse primers used for amplification were *PXR8055C > T* (forward: 5′-GGCAGGAAGATGGAATGG-3′ and reverse: 5′-GGGAGAAGAGGGAGATGG-3′). The 50 μl reaction mixture included 25 μl of 2 × Taq PCR Master Mix (Applied Biosystems), 1 μl of each of primers, 0.5 μl of cDNA template, and 22.5 μl of deionized water (diH_2_O). The amplification of *PXR8055C > T* was performed by the following process: 3-min initial denaturation at 94 °C, denaturation at 94 °C for 30s, anneal at 57 °C for 30s, elongation at 72 °C for 30s, followed by 30 cycles, then extended at 72 °C for 5 min. The amplification products were kept at 4 °C and identified by the agarose gel electrophoresis. Genotyping of SNPs was performed by direct sequencing. For each polymorphism of *PXR (PXR11156A > C*, *PXR11193T > C and 8055 C > T)*, patients were assigned to three groups according to the genotypes: wild homozygotes, heterozygotes group and mutant homozygotes. Patients were also divided into three haplotypes analyzed by PHASE V.2.1 software: *non-PXR* ^*^ *1B* haplotype, *PXR* ^*^ *1B* haplotype and *PXR* ^*^ *1B/* ^*^ *1B* haplotype as described in the background (Table 1).

**Table 1:** Composition of *PXR* polymorphisms and *PXR* ^*^ *1B* haplotypes

	*Non-PXR* ^*^ *1B* (*n* = 73)	*PXR* ^*^ *1B* (*n* = 140)	*PXR* ^*^ *1B/* ^*^ *1B* (*n* = 70)
*1156 A > C*	*AA* (73)	*AC* (140)	*CC* (75)
*11193 T > C*	*TT* (75)	*TC* (141)	*CC* (71)
*8055 C > T*	*CC* (74)	*CT* (144)	*TT* (70)

Data are expressed as numbers

### Evaluation of CYP3A4 activity

The CYP3A4 activity was determined by the plasma ratio of 1′-hydroxymidazolam (1′-MDZ) to midazolam (MDZ), at 1 h after the intravenous injection of midazolam 0.1 mg/kg for induction. Liquid chromatography mass spectrometry (LC/MS) was used to measure the level of 1′- MDZ and MDZ as described by Kanazawa et al. [16].

### Statistical analysis

SPSS 17.0 software (SPSS Inc., Chicago, Illinois, USA) was used for statistical analyses. Values were reported as $mean\pm SD$. The Chi-square test was used to verify Hardy-Weinberg equilibrium. Data for the fentanyl consumption were compared using one-way analysis of variance with post hoc Bonferroni correction. Between-group comparisons of the VAS score were performed with the Mann–Whitney *U* test. Multiple comparisons were performed before and after being adjusted for age, weight and the intraoperative remifentanyl dosing. The incidences of adverse effects were analyzed using the Chi-square test or Fisher exact test. Power analysis of the post-operative fentanyl consumption was performed with the NCSS PASS 2008 (NCSS Inc., Kaysville, Utah, USA). A *P*-value of less than 0.05 was set to indicate statistical significance.

## Results

All 287 women who were taken from the larger cohort were included in the final pain analysis final. The frequency of the *PXR11193T > C* allele was 49.6%; the frequency of *PXR8055C > T* allele was 49.3%; the frequency of *PXR* ^*^ *1B* haplotype was 48.8%. Allele frequencies were in Hardy-Weinberg equilibrium (*P* > 0.05).

Although patients received an intravenous infusion of fentanyl (1 μg/kg) approximately 30 min prior to the completion surgery, the VAS pain score at the end of surgery was a mean of 6.1 ± 1.2. The pain score At 24 h after surgery was 2.2 ± 0.7 on average. No patient required rescue management for inadequate pain control. The mean fentanyl consumption was 452 μg ± 86 μg in the 24 h period after surgery. The incidence of postoperative nausea and vomiting was 20.8%. The incidence of mild sedation and dizziness in our study was 6.9 and 5.6%, respectively.

There was no difference in demographics, VAS pain scores at the end of surgery or at 24 h postoperatively between groups (*P* > 0.05). There were no statistically significant differences in PCA fentanyl consumption for the 24 h period after surgery between groups (*P* > 0.05). Additionally, the enzyme activity of CYP3A4 showed no significant variation relative to genotype (Tables 2 and 3). There were no statistical differences in the incidence of the side effects between PXR haplotypes (*P* > 0.05)

**Table 2:** General information for *PXR* ^*^ *1B* haplotypes

	*Non-PXR* ^*^ *1B*	*PXR* ^*^ *1B*	*PXR* ^*^ *1B/* ^*^ *1B*	*P* value
Age (years)	41 ± 5	40 ± 6	42 ± 4	0.100
BMI (kg/m^2^)	21.8 ± 2.0	22.1 ± 2.1	23.0 ± 2.3	0.666
Total hysterectomy	50	92	39	0.832
Myomectomy	22	48	21	0.224
Operation time (min)	90 ± 14	88 ± 17	95 ± 11	0.804
Propofol (mg)	560 ± 89	550 ± 90	570 ± 91	0.770
Remifentanil (μg)	921 ± 130	914 ± 106	945 ± 137	0.449

Data are expressed as numbers or mean ± SD. Drug doses are totals

**Table 3:** Effect of *PXR* ^*^ *1B* haplotypes on postoperative analgesia

	*Non-PXR* ^*^ *1B*	*PXR* ^*^ *1B*	*PXR* ^*^ *1B/* ^*^ *1B*	*P* value
Initial postoperative VAS scores	6.1 ± 1.3	6.2 ± 1.1	5.9 ± 1.0	0.414
Mean VAS scores during first 24 h	2.1 ± 0.7	2.3 ± 0.8	2.2 ± 0.8	0.601
Fentanyl consumption during first 24 h (μg)	468 ± 83	453 ± 85	435 ± 87	0.062
^b^CYP3A activity (1′-OHMDZ:MDZ)	0.40 ± 0.12	0.39 ± 0.14	0.40 ± 0.11	0.885

Data are expressed as mean ± SD. Statistical significance was set at *P* < 0.05. *P* values are adjusted by age, sex, weight, remifentanyl dose during the operation, and type of surgery. ^b^CYP3A4 activity (*n* = 95)

## Discussion

PXR is a key transcription activating factor for the expression of the CYP3A4 gene [13, 14]. A model of transgenic mice, produced in 2000, demonstrated that PXR plays an important role in the expression of CYP3A4 [13]. The *PXR* ^*^ *1B* haplotype (*PXR1156A > C, PXR11193T > C* and *PXR8055C > T)* has been shown previously to effect the metabolic parameters of doxorubicin in patients with breast cancer [17]. Gender has been reported to influence the enzyme activity of CYP3A4 [18], however, since this study was limited to gynecology patients, gender variation was not an issue.

Midazolam was used as a probe drug for the CYP3A4 enzyme and is metabolized to 1′-OH midazolam by CYP3A4 enzyme. The metabolic breakdown of midazolam is primarily influenced by the activity of CYP3A4, not blood flow to the liver [19]. However, we found no significant differences in metabolism based on genotype.

We noted that occurrence rate of *PXR* ^*^ *1B* was 48.8% and appeared to be in Hardy-Weinberg equilibrium.

Our study demonstrated that the *PXR* ^*^ *1B* haplotype did not influence the analgesic effects of fentanyl postoperatively and therefore does not appear to be a main determining factor for variations in fentanyl use.

In Asian patients with breast cancer, *PXR* ^*^ *1B* haplotype affected the *PXR* mRNA level. Patients with the PXR*1B haplotype showed a lower *PXR* mRNA level compared with the patients with non- PXR*1B haplotype. PXR*1B-bearing liver tissues were associated with significantly lower expression of CYP3A4 compared with non- PXR*1B-bearing liver tissues. Furthermore, *PXR* ^*^ *1B* haplotype had an reducing-effect on the elimination rate of doxorubicin [19, 20]. In the present research, difference of the fentanyl consumption during the first 24 h postoperatively among the *PXR* ^*^ *1B* haplotypes was not found*.* However, a decreasing trend of the fentanyl consumption during the first 24 h postoperatively among the *PXR* ^*^ *1B* haplotypes was found. That was, fentanyl consumption was much lower in the patients containing *PXR* ^*^ *1B* haplotype. It might due to the limitation of our research sample size, statistical significance could not be obtained. Since it is impractical to obtaine liver samples from the subjects. The CYP3A4 protein levels were not be able to be detected, either. On the other hand, doxorubicin and fentanyl are also substrates of P-glycoprotein encoded by ABCB1 gene. Since P-glycoprotein is also affected by the nuclear receptor-PXR at transcriptional level [21], we speculate that the genetic variations of the *PXR* ^*^ *1B* haplotype have a more significant effect on *ABCB1* than *CYP3A4.*

The study only evaluated the effects of the *PXR* ^*^ *1B* haplotype on the analgesic effect of fentanyl. The determination of the fentanyl metabolism in vivo may be impacted by not only the protein expression, but also the CYP3A4 activity, and other factors such as CYP3A5 enzyme and other drug interactions etc. Due to study limitations we were unable to actually determine the level of CYP3A4 protein expression in the hepatocytes of subjects. In the future a study that extracts microsomes from hepatic cells and assesses the protein expression and the activity of CYP3A4 enzyme in vitro may avoid any confounding factors.

There was a limitation to our research. 330 samples were collected during 2007 and 2010. Although we almost genotyped the entire DNA samples, not every sample had the whole data we need in this research. Especially for the CYP3A enzyme activity, we only got 95 samples’ data. The statistical power for the fentanyl consumption was 0.736 and it was low for the CYP3A enzyme activity.

## Conclusions

In summary, we investigated the effects of the polymorphisms of *PXR* ^*^ *1B* on intravenous postoperative analgesia with fentanyl in patients undergoing selective abdominal total hysterectomy or myomectomy under general anesthesia. Our results failed to found significant effects contributed by the polymorphisms of *PXR* ^*^ *1B*.

## Abbreviations

1′-MDZ: 1′-hydroxymidazolam
ASA: American Society of Anesthesiologists
CYP450: Cytochrome P450
LC/MS: Liquid chromatography mass spectrometry
MDZ: Midazolam
mRNA: Messager RNA
PCA: Patient-controlled analgesia
PCIA: Patient-controlled intravenous analgesia
PCR: Polymerase chain reaction
*PXR*: Pregnane X Receptor
SNP: Single nucleotide polymorphism
VAS: Visual analog score
